# Anteroposterior chest radiograph vs. chest CT scan in early detection of pneumothorax in trauma patients

**DOI:** 10.1186/1755-7682-4-30

**Published:** 2011-09-27

**Authors:** Hesham R Omar, Devanand Mangar, Suneel Khetarpal, David H Shapiro, Jaya Kolla, Rania Rashad, Engy Helal, Enrico M Camporesi

**Affiliations:** 1 Departement of Internal Medicine, Mercy Hospital and Medical Center. Chicago, Illinois, USA; 2 Department of Anesthesiology; Tampa General Hospital; Tampa, Florida, USA; 3 Florida Gulf to Bay Anesthesiology, Tampa, Florida, USA; 4 Department of Surgery, University of South Florida, Tampa, Florida, USA; 5 Department of Critical Care Medicine, Cairo University Hospital, Cairo, Egypt; 6 Emergency Department, Elagouza Hospital, Cairo, Egypt; 7 Department of Surgery/Anesthesiology, University of South Florida, Tampa, Florida, USA

## Abstract

Pneumothorax is a common complication following blunt chest wall trauma. In these patients, because of the restrictions regarding immobilization of the cervical spine, Anteroposterior (AP) chest radiograph is usually the most feasible initial study which is not as sensitive as the erect chest X-ray or CT chest for detection of a pneumothorax. We will present 3 case reports which serve for better understanding of the entity of occult pneumothorax. The first case is an example of a true occult pneumothorax where an initial AP chest X-ray revealed no evidence of pneumothorax and a CT chest immediately performed revealed evidence of pneumothorax. The second case represents an example of a missed rather than a truly occult pneumothorax where the initial chest radiograph revealed clues suggesting the presence of pneumothorax which were missed by the reading radiologist. The third case emphasizes the fact that "occult pneumothorax is predictable". The presence of subcutaneous emphesema and pulmonary contusion should call for further imaging with CT chest to rule out pneumothorax. Thoracic CT scan is therefore the "gold standard" for early detection of a pneumothorax in trauma patients. This report aims to sensitize readers to the entity of occult pneumothorax and create awareness among intensivists and ER physicians regarding the proper diagnosis and management.

## Introduction

The concept of occult pneumothorax is well accepted among the surgical trauma literature [1-5]. In trauma patients, because of restrictions regarding cervical spine immobilization, AP chest radiograph is usually utilized to detect intrathoracic pathology. This report emphasizes how AP chest radiograph can dangerously delay the recognition of a pneumothorax. More advanced imaging modalities including Chest CT scan or ultrasonography is therefore manadatory to exclude the diagnosis.

## Case # 1

**Figure 1:**
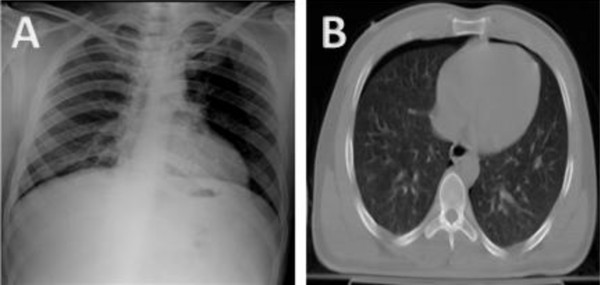
**Anteroposterior chest X-ray and CT scan chest demonstrating "true occult pneumothorax"**. Anteroposterior chest X-ray revealing no evidence of pneumothorax (Panel A). CT chest performed immediately after X-ray revealing right sided pneumothorax (Panel B). Adapted from Omar et. al. [6].

A 24-year-old male presented to the ER after a motor vehicle accident. On admission the patient was confused with a Glasgow coma score of 14/15. CT brain revealed brain edema and fracture skull base. Chest exam and arterial blood gases were satisfactory. AP chest X-ray revealed no evidence of pneumothorax as demonstrated in Figure 1 panel A, adapted from Omar et. al. [6] CT chest performed immediately after the X-ray revealed a right sided pneumothorax (Figure 1 panel B), adapted from Omar et. al [6]

This case represents an example of a true occult pneumothorax where an AP chest X-ray failed to show an existing pneumothorax. This emphasizes the importance of chest CT in any trauma victim who is tachypnic or hypoxic when the initial AP chest radiograph appears normal. This is especially important in patients expected to be maintained on positive pressure ventilation.

## Case # 2

**Figure 2:**
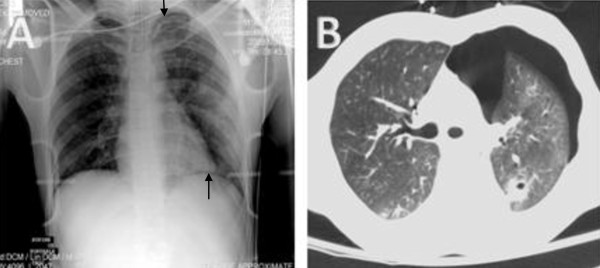
**Anteroposterior chest X-ray and CT scan chest demonstrating "missed pneumothorax"**. Initial Anteroposterior chest X-ray of the intubated patient, illustrating diffuse air space opacities in the left lower lung field. Underlying pneumothorax was suggested because of a visible pleural stripe in the lung apex and a highly visible cardiophrenic sulcus (Panel A). Chest CT scan illustrating a left-sided pneumothorax with underlying lung collapse (Panel B). Adapted from Omar et. al. [6].

A 29-year-old restrained driver was involved in a T-bone vehicular accident. At the scene of the accident the patient's Glasgow coma score was 4/15. The patient was intubated for airway protection and sent to the ER. While in the ER, an AP chest X-ray was completed (Figure 2 panel a), adapted from Omar et. al, [6] which revealed a mechanically ventilated patient with diffuse airspace opacities prominently located in the left lower lung field. In the setting of trauma, this was interpreted as lung contusions.

The patient was immediately sent for a chest CT scan (Figure 2 panel b), adapted from Omar et. al, [6] that was performed 15 minutes after the chest X-ray and revealed a large left sided pneumothorax, a left lower lobe collapse as well as hemorrhagic contusions of both lower lobes. The anesthesiology/critical care team then decided to insert a chest tube which was followed by relief of the pneumothorax and lung re-expansion.

This case represents an example of a missed rather than a truly occult pneumothorax. The presence of a visible pleural stripe at the lung apex and a highly visible cardiophrenic sulcus should have drawn attention to an underlying pneumothorax. This case emphasizes the importance of interpretation of the X-ray both by an experienced radiologist as well as the managing trauma team to avoid missing a pneumothorax.

## Case # 3

**Figure 3:**
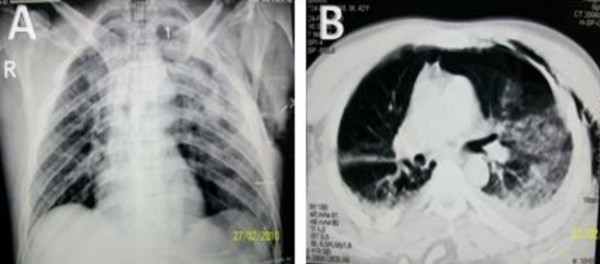
**Anteroposterior chest X-ray and CT scan chest demonstrating the predictability of occult pneumothorax**. AP chest X-ray revealing evidence of bilateral lung contusions and left subcutaneous emphesema (Panel A). Chest CT confirming both the lung contusions and the subcutaneous emphesema and demonstrating left sided pneumothorax not initially appearing on the anteroposterior chest X-ray (Panel B). Adapted from Omar et. al.[6].

A 42-year-old male presented to the ER after a motor vehicle accident. On admission, his Glasgow coma score was 7/15 and CT brain revealed a left thalamic hemorrhage, subarachinoid hemorrhage and brain edema. Chest examination revealed equal air entry on both sides with coarse ronchi and crepitations over the left hemithorax. There was clinical evidence of subcutaneous emphesema in the left chest wall. The patient was hypoxic on room air and an arterial blood gases revealed a PH of 7.35, PO2 66.4 mmHg, PCO2 33.4 mmHg, HCO3 18.2 and SO2 91.4% so the patient was maintained on supplemental oxygen of 10 liters/minute supplied by a face mask. AP chest X-ray revealed pulmonary contusions more on the left lung and left sided subcutaneous emphesema with no clear evidence of pneumothorax (Figure 3 panel a), adapted from Omar et. al. [6] Chest CT scan immediately performed revealed left sided pneumothorax and an intercostal tube was inserted, (Figure 3 panel b), adapted from Omar et. al, [6]

This case illustrates the predictability of occult pneumothorax. The presence of subcutaneous emphesma and pulmonary contusions highly predicts for the presence of an underlying pneumothorax. We therefore suggest that any trauma victim presenting with subcutaneous emphesma, pulmonary contusion or rib fractures should be further investigated with a CT chest if the chest X-ray did not reveal pneumothorax. This is especially important in patients who will receive mechanical ventilation for fear of development of tension pneumothorax.

## Discussion

The widespread availability and utilization of CT scan in the evaluation of trauma patients accounted for the diagnosis of pneumothoraces that were not initially evident on the chest X-ray. The reported incidence of occult pneumothorax varies between 3.7% in injured children presenting to an emergency department to 64% in intubated multi-trauma patients [7-9]. The proportion of pneumothoraces that are occult compared with those actually seen on the chest X-ray ranges from 29% to 72% [1-4,10,11]. Most instances of occult pneumothoraces in the supine patient are anterior and usually located in the superior thorax or thoracic apex indicating that the gold standard for ruling out occult pneumothorax is a thoracic CT scan. Occult pneumothorax is more concerning in trauma patients who have diminished cardiopulmonary reserve because of the risk of rapid progression to tension pneumothorax especially those on positive pressure ventilation.

The traditional management of post-traumatic pneumothoraces has been the placement of a chest tube [5]. The choice between close observation vs. early intercostal tube placement is still unsettled. In patients who are asymptomatic, not mechanically ventilated, with a pneumothorax that is not increasing in size, clinical opinion supports that close observation is safe [3,4,12,13]. However, some authors believe that the risk of progression to tension pneumothorax is significant and that prophylactic chest tube placement is necessary for any patient subjected to positive-pressure ventilation is necessary [10,14,15]. Of note is that the size of the initial occult pneumothorax does not predict for the development of tension pneumothorax and therefore should not be used as a guide for chest tube placement [16].

The 3 case presentations well illustrated the entity of occult pneumothorax. The first case is a classic example of a true occult pneumothorax which did not show initially on the AP chest X-ray and was diagnosed by chest CT scan. Missing the diagnosis is extremely dangerous for fear of the development of tension pneumothorax especially if the patient is placed on a mechanical ventilator. The second case is not a true occult pneumothorax and is better nominated as a missed pneumothorax where the X-ray report did not confirm the presence of pneumothorax when there were 2 clues for the diagnosis. The presence of an apical pleural stripe and a clearly visible cardiophrenic sulcus should have drawn attention to the diagnosis. We therefore suggest that the X-ray should be interpreted by a well trained radiologist and also by the treating physician who, in the setting of a trauma patient, will have a higher index of suspicion for the diagnosis. The third case demonstrated the predictability of occult pneumothorax. The 3 factors that have a predictive value for occult pneumothorax are the presence of subcutaneous emphesema, lung contusions and rib fractures [17]. In this patient the presence of subcutaneous emphesema directed our attention for further imaging looking for a pneumothorax despite its absence on the AP chest radiograph. It should also be noted that the surgical emphesema can obscure the underlying pleural air further limiting the accuracy of the Chest X-ray in diagnosing pneumothorax in such cases.

We conclude that thoracic CT scan is the "gold standard" for early detection of a pneumothorax in blunt trauma patients. In centers utilizing eFAST (Extended Focused Assessment with Sonography for Trauma) technology for trauma victims, this should be the initially utilized modality because of its high sensitivity, no need for transporting an unstable patient and its low radiocarcinogenesis profile.

## Consent

Written informed consent was obtained from the patient's relatives for publication of this article. A copy of the written consent is available for review by the Editor-in-Chief of this journal

## Competing interests

The authors declare that they have no competing interests.

## Authors' contributions

HO is responsible for literature search and drafting the manuscript and providing the explanatory figures. JK, EC and DM, RR, DS, SK and EH have made critical revisions to the manuscript. All authors have read and approved the whole manuscript.
